# Immunoinformatics‐Based Multi‐Epitope Vaccine Design Against *P. falciparum‐*Causing Malaria: A Computational Approach

**DOI:** 10.1002/hsr2.71353

**Published:** 2025-10-09

**Authors:** Amma Aboagyewa Larbi, Caleb Mensah, Rebecca Korankye, Andrew Appiah Darkwah, Esther Boakye Agyemang, Isaac Yao Ayikpah, Alexander Kwarteng

**Affiliations:** ^1^ Department of Biochemistry and Biotechnology Kwame Nkrumah University of Science and Technology Kumasi Ghana; ^2^ Kumasi Center for Collaborative Research in Tropical Medicine (KCCR) Kumasi Ghana

**Keywords:** epitopes, immunoinformatics, malaria

## Abstract

**Background and Aim:**

Malaria is an infectious disease that affects people in Africa, and other parts of the world by the millions. Despite the effectiveness of artemisinin‐based combination therapies against the disease, current findings on possible *Plasmodium* resistance to artemisinin warrants an exploration of new treatment options. This study aimed to design a multi‐epitope vaccine against *Plasmodium falciparum* (*P. falciparum*) using immunoinformatics and computational techniques.

**Methods:**

In this study, we employ immunoinformatics and computational techniques to design a multi‐epitope vaccine against *P. falciparum*. B cell epitopes, cytotoxic T (Tc) cell and helper T (Th) cell epitopes were identified from five highly conserved *P. falciparum* antigens that play crucial roles in the pathogenicity of the infection. Predicted epitopes were selected based on set criteria and concatenated with appropriate adjuvants and linkers to construct a multi‐epitope subunit vaccine.

**Results:**

Collectively, the Tc cell and Th cell epitopes of the proposed vaccine cover an estimated 87.07% of the world′s population, based on the HLA data set from the Allele Frequency Net Database. The vaccine construct was found to be soluble and have appropriate physicochemical properties. The docking, and molecular dynamics (MD) simulation studies carried out revealed strong and stable interactions between TLR4 and the vaccine construct. Immune simulation upon administration of the vaccine construct indicates the development of long‐lasting memory B, and CD4+ T cells for antigen clearance.

**Conclusion:**

This computational study presents a promising multi‐epitope subunit vaccine candidate against P. falciparum. The construct shows high population coverage, stability, and potential immunogenicity. These findings however require further experimental validations.

## 1 Introduction

Malaria continues to be one of the deadliest infectious diseases known to man despite a plethora of global and modern initiatives aimed at reducing its public health outcomes. This disease is a potential life‐threatening infection with not only significant morbidity but also devastating economic consequences, especially in developing countries [1, 2]. According to the World Malaria Report, 2023 [3], 2020 alone recorded an estimation of 608,000 people dying of malaria, and 249 million new malaria cases worldwide. Of these statistics, Africa records the highest burden of all malaria cases, registering close to 94% of all clinical cases, and 95% of its global mortalities. This highlights the need to identify effective antimalaria treatment options to mitigate the mortality burden of malaria across Africa.

This disease is caused by *Plasmodium* parasites which are transmitted by female *Anopheles* mosquitoes. *Plasmodium falciparum* (*P. falciparum)* and *P. vivax* are the most prevalent forms of the parasite, with *P. falciparum* seen as the most lethal among the species [4]. The *Plasmodium* parasite is primarily transmitted into the bloodstream of humans by the bite of a *Plasmodium*‐infested female *Anopheles* mosquito, however, transfusion of infected blood or congenital transmission can also bring about the malaria infection [5]. The life cycle of the parasite is constituted by four stages namely liver stage, blood stage, and transmission stage in its human host, and the final stage being the mosquito stage [6].

Currently, malaria can be treated with a fixed dose of oral artemisinin‐based combination therapies (ACTs) medications. The most widely used combination therapies are Artemether‐lumefantrine, and amodiaquine‐artesunate [7]. However, with recent emergence of resistance to artemisinin by the *Plasmodium* parasite [8], there is the need to develop novel strategies to prevent and treat malaria disease.

Vaccines are one explorative avenue for the prevention and treatment of malaria. RTS,S/AS01 (Mosquirix; GlaxoSmithKline) is the first‐ever malaria vaccine that has been recommended by the World Health Organization [9]. Although promising, the Malaria Vaccine Implementation Program revealed that the recommended malaria vaccine only confers about 30% reduction in cases of severe malaria [10]. However, for the WHO goal of supplying a malaria vaccine candidate with a 75% or greater efficacy against clinical malaria to be achieved by 2030, there is need to identify more novel and efficacious malaria vaccines [11].

The use of computational approach to design epitope‐based vaccines presents a comparatively cost effective, and faster approach compared to conventional approaches. These peptide‐based vaccines reduce the burden of antigenic load and allergy induction that is seen in most vaccines of conventional designs. Also, because of their multi‐epitope design, multi‐epitope vaccines can be engineered to elicit cell‐mediated, and humoral immunological responses due to the incorporation of B‐cell, cytotoxic T cell, and helper T cell epitopes, respectively in the construct [12].

*Plasmodium* parasites have developed various mechanisms to evade the immune system of the host. One such mechanism is antigenic variation, where the parasite switches the expression of variant surface antigens to escape recognition by the host immune response [13, 14, 15]. However, some antigens, like PfTRAP, PfRH5, PfSEA‐1, PfGARP, and PfCyRPA, are relatively conserved across different strains of *Plasmodium* [16, 17, 18]. This conservation may be due to their importance in critical stages of the parasite life cycle, making them less prone to variation and ensuring their functional integrity. Again, the conserved nature of these antigens may be due to functional constraints imposed by their specific roles. For example, PfRH5 is involved in binding to a receptor on the host red blood cell surface, and any significant changes in its structure could disrupt this interaction. Similarly, PfTRAP plays a role in gliding motility and invasion, and hence altering its structure could affect the parasite′s ability to invade host cells. These functional constraints contribute to the conservation of these antigens [19]. This study employs the use of bioinformatics to identify a multi‐epitope vaccine that targets these conserved antigens found in *P*. falciparum. Our results indicate that the proposed multi‐epitope vaccine can potentially induce high B cell and Th1‐specific immunity against the selected malaria parasite.

## 2 Methods

**Figure 1:**
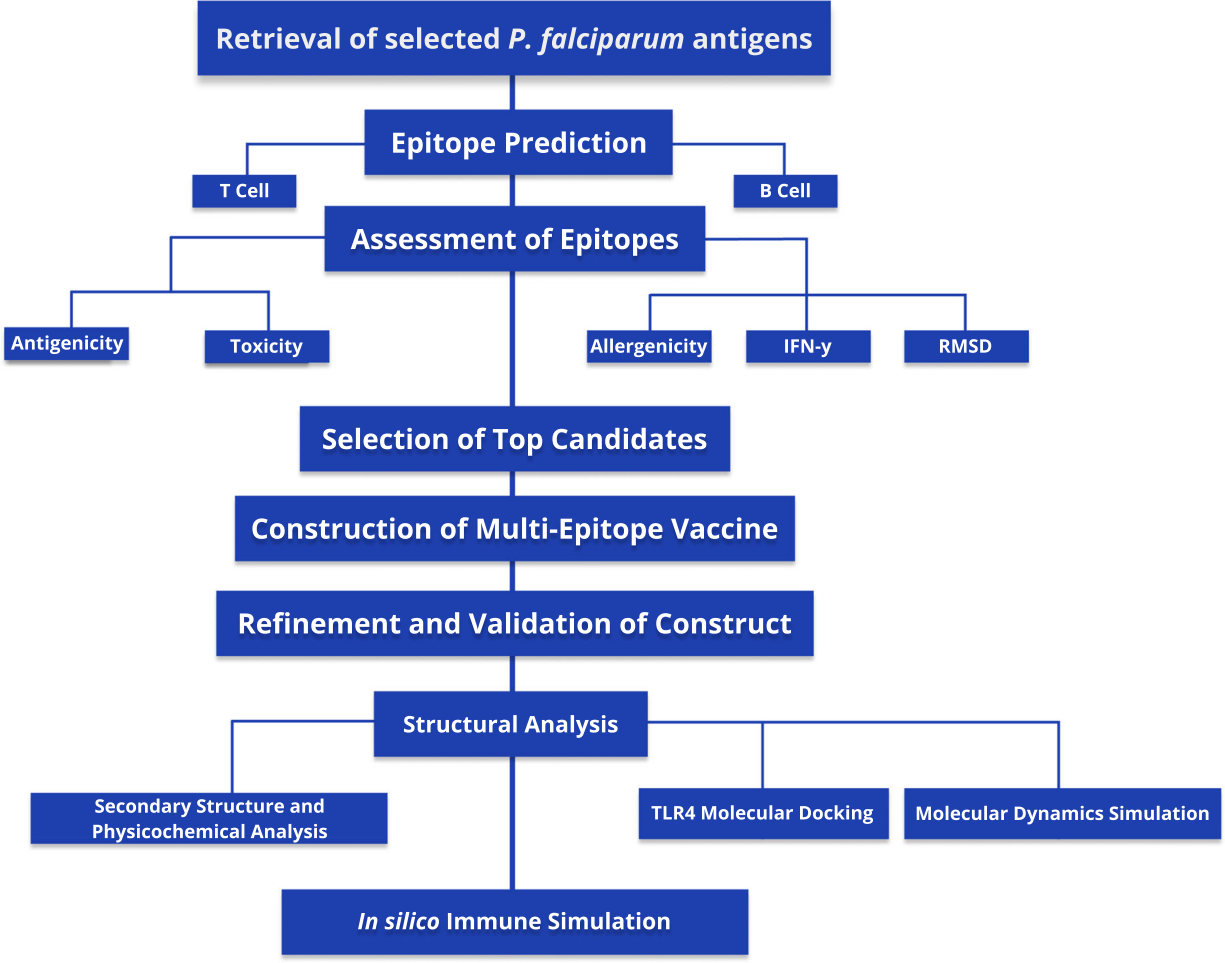
Workflow of the study design used for the vaccine construct. Ideal B, and T cell epitopes were identified using a series of prediction tools, based on set selection criteria for assessment, and used to construct the vaccine candidate for further analyzes. This was followed by molecular docking, dynamics simulation, and simulation of immune responses upon administration.

The immunoinformatics based workflow for this study is shown in Figure 1.

### 2.1 Retrieval of Selected Plasmodium Antigen's Sequences

The amino acid sequence of the *Plasmodium* antigens explored in this study; PfTRAP, PfRH5, PfSEA‐1, PfGARP, and PfCyRPA, were retrieved from the UniProt database (https://www.uniprot.org), with their respective entry identifiers being Q94662, Q8IFM5, A0A143ZXM2, P13816, and Q8IFM8.

### 2.2 Prediction and Assessment of Epitopes from Target Antigens

#### 2.2.1 B‐cell Epitopes Prediction

Cell surface receptors of B‐cell lymphocytes recognize potential B‐cell epitopes. The characterization of these epitopes leads to the production of a specific humoral response. They play a key role in the efficiency of vaccines [20]. In this study, the Bepipred server (https://services.healthtech.dtu.dk/services/BepiPred-2.0/) was utilized to predict flexible length linear B‐cell epitopes in the protein sequence, and the ElliPro server (http://tools.iedb.org/ellipro/) was used to predict discontinuous epitopes. For ElliPro inputs, the 3D structures of the target antigens were modeled using AlphaFold v2.0 (https://github.com/deepmind/alphafold).

#### 2.2.2 T Cell Epitopes Prediction

Immunological studies and vaccine development are crucial for the characterization of peptide epitopes that are restricted to MHC class binding epitopes. In this study, the protein amino acid sequences were submitted to the Immune Epitope Database (IEDB) web server at http://tools.iedb.org, to predict MHC class I and II peptide binding epitopes. This server utilizes an artificial neural network of algorithms and classifies weak and strong binders to each HLA (HLA‐A, HLA‐B, HLA‐C; HLADR, HLA‐DP, and HLA‐DQ) allele based on their percentile rank and the affinity of peptides. Thus, a peptide with a high affinity has a low percentile rank [21, 22].

#### 2.2.3 Epitope Assessment

All predicted B‐cell epitopes were assessed based on their antigenicity, allergenicity, and toxicity whiles predicted T‐cell epitopes were evaluated based on antigenicity, allergenicity, toxicity, and their ability to induce interferon‐gamma. The Vaxijen server (https://www.ddg-pharmfac.net/vaxijen/VaxiJen/VaxiJen.html) was used for evaluating the probable antigenicity of the epitopes. This method utilizes an alignment‐independent algorithm that classifies antigens based solely on the physicochemical properties of proteins without regard to sequence alignment [23]. In this study, the parasite model was set at a threshold of 0.5 for assessment.

The Allergenicity was assessed using AllerTop v.2.0; a server that works based on different methods including auto cross‐covariance transformation, k nearest neighbor (kNN), machine learning, and amino acid E‐descriptors for classifying allergens [24]. This server can be accessed via the URL at https://www.ddg-pharmfac.net/AllerTOP/. The toxicity of the predicted epitopes was evaluated using a server called ToxinPred (https://crdd.osdd.net/raghava/toxinpred/). The server allows for the prediction and designing of toxic and nontoxic peptides [25]. It employs a support vector machine‐based algorithm.

The predicted T‐cell epitopes were screened for IFN‐gamma induction using the IFN epitope server (http://crdd.osdd.net/raghava/ifnepitope/index.php. The prediction was done with the hybrid approach based on motif and SVM and IFN‐gamma versus non‐IFN‐gamma model [26]. Only peptides of epitopes that met the criteria for being a probable antigen, nontoxic, probable non‐allergen, and positive to induce IFN‐gamma were selected for further experiments.

#### 2.2.4 Molecular Dynamics Simulation of Selected Epitopes

The selected peptides were subjected to molecular dynamics simulation. Molecular dynamic simulation is key to understanding the behavior of biomolecules [27]. It is a known approach used to study the structural and functional properties of macromolecules at the microscopic level. Google Collaboratory cloud space (https://colab.research.google.com), based on Jupyter Notebooks, utilizes OpenMM engine and AMBER force field to run the simulation [28]. It gives a runtime fully configured for deep learning and grants free‐of‐charge access to a robust GPU [29]. All dependencies were installed, and the Google service was linked to a Google Drive account for storage of all simulation data. PDB files of the selected epitopes were then loaded as input files on the OpenMM engine, a molecular dynamics package for running simulations. In this study, a topology was successfully generated using the Assisted Model Building with Energy Refinement (AMBER) program applied together with its various models. The force field used for the complex was ff19SB and the system was solvated in a three‐point transferable intermolecular potential (TIP3P) water model with size dimensions of 12 angstroms. The system was neutralized with 0.15 M NaCl, coupled with a 10,000‐step energy minimization of maximum force threshold of 100 kJ/mol to remove any clash or steric hindrance on the system. The system was then subjected to NPT equilibration at 298 K and 1 bar for 0.5 ns/500 ps with an integration time of 2 fs, followed by a 100 ns production simulation using the equilibrated system coordinates as input structure. At the end of the production simulation, the strides were concatenated to form a complete trajectory file which was visualized and analyzed. The root mean square deviation (RMSD) values of the alpha carbon atoms were computed. RMSD value compares the relative position of protein alpha carbons by computing the mean of amino acids in the 3‐dimensional space.

### 2.3 Multi‐Epitope Vaccine Construction, Modeling, Refinement, and Validation of Vaccine Construct

The immunoinformatics approach, which combines computational and molecular immunological tools, can be used to identify target antigens for vaccine development [30].

By the combined effect of T‐cell epitopes, B‐cell epitopes, and adjuvants with appropriate linkers, the multi‐epitope subunit vaccine candidate can be constructed. All selected epitopes that met our selection criteria were considered for the vaccine construct. These epitopes were joined with ideal peptide linkers and adjuvants. The B cell epitopes, MHC‐I and MHC‐II epitopes were linked using KK, AAY, and GPGPG linkers, respectively. Studies have revealed that TLR2 and TLR4 can recognize *P. falciparum* glycosylphosphatidylinositol [31]. Hence, adjuvants used in this study were the TLR‐4 adjuvant RS‐09 (sequence: APPHALS), and TLR2 adjuvant human β‐defensin‐3 (sequence: GIINTLQKYYCRVRGGRCAVLSCLPKEEQIGKCSTRGRKCCRRKK). These adjuvants were placed at the N‐terminal of the vaccine construct and linked with the rigid EAAAK linker. Finally, the TAT sequence (TGALLAAGAAA) was placed at the C‐terminal of the vaccine to enable intracellular delivery of the vaccine construct [32]. With the adjuvants and the TAT sequence respectively situated at the N‐. and C‐terminal of the vaccine, the predicted B cell epitopes, MHC‐I epitopes, and MHC‐II epitopes were shuffled in between the N‐ and C‐terminal resulting to six different models of the vaccine (i.e; vac1 to vac6).

### 2.4 Modeling, Refinement and Validation of Vaccine Construct

The 3D structures of the six vaccine constructs were modeled using AlphaFold v2.0 (https://github.com/deepmind/alphafold). These 3D structures were then refined using GalaxyRefine web server (https://galaxy.seoklab.org/cgi-bin/submit.cgi?type=REFINE). The server rebuilds and repacks all side‐chain conformations, which is followed by a repeated relaxation of the structure achieved through molecular dynamics simulation [33]. Among the six‐vaccine constructs, the best refined 3D model based on the server′s predicted RMSD values, was further validated using ProSA‐web (https://prosa.services.came.sbg.ac.at/prosa.php) and ProCheck (https://saves.mbi.ucla.edu/). The ProSA‐web server compares the inputted protein structure with the X‐ray crystallography and NMR values of experimentally validated protein structures to predict an overall quality score. Quality scores falling outside the given range of native proteins indicate possible errors in the protein structure inputted [34]. The ProCheck server examines the stereochemical quality of a protein structure by analyzing individual residue geometry and overall structure geometry to ascertain the overall quality of the structure [35]. The Ramachandran plot, and Z‐score were obtained from the ProCheck and ProSA web servers respectively.

### 2.5 Population Coverage Analysis

Various human leukocyte antigen (HLA) alleles are expressed in diverse frequencies in different geographical and ethnic backgrounds. Hence for a successful multi‐epitope based vaccine development, it is important to characterize the HLA‐alleles distribution among the world population [36]. To assess the population coverage of the vaccine candidates, the selected MHC‐I and MHC‐II epitopes, as well as their respective HLA‐alleles were inputted into the IEDB population coverage analysis tool (http://tools.iedb.org/population/). This tool employs the robust HLA data set that captures allele frequencies from multiple global subpopulations. [37, 38] The total population coverage was carried out globally and for multiple African regions.

### 2.6 Secondary Structure, Physicochemical Analysis, and Solubility Prediction of Vaccine Construct

The secondary structure of the vaccine construct was generated using PSIPRED63 (http://bioinf.cs.ucl.ac.uk/psipred). The antigenicity, toxicity, allergenicity, and other physicochemical properties such as the nature and the stability of the vaccine construct were also determined. The nature and the stability of the vaccine construct were determined using the ProtParam server (https://web.expasy.org/protparam/). The solubility of the vaccine construct was predicted using the Protein‐Sol server (https://protein-sol.manchester.ac.uk/).

### 2.7 Molecular Docking Simulation of TLR4 and Vaccine Construct

To determine the binding potency between the vaccine construct and TLR4 (PDB ID: 3FXI), Cluspro 2.0, a server (https://cluspro.bu.edu/ that uses PIPER (a fast frontier transform‐based algorithm) to perform a protein‐to‐protein docking was used. The ClusPro server performs rigid‐body docking of two interacting proteins using a Fast Fourier Transform‐based algorithm called PIPER to sample numerous conformations. Following this, it clusters the 1000 lowest energy structures obtained based on their RMSD values, and refines these selected structures using energy minimization [39, 40]. The docked structure with the most cluster members having the lowest energy was visualized with PyMol (https://pymol.org/2/) to analyze the interactions between TLR4 and the vaccine.

### 2.8 Molecular Dynamics Simulation of TLR‐Vaccine Complex

Molecular dynamics (MD) simulation of the TLR‐vaccine complex was performed using WebGro (https://simlab.uams.edu/). Briefly, the best pose of the receptor‐vaccine complex was inputted into the “protein in water simulation” tool of WebGro. The forcefield for MD simulation was set to GROMOS96 43a1. Following this, SPC was chosen as the water model, and the box type set to “Triclinic.” The system was then neutralized with 0.15 M of sodium chloride (NaCl) before a 5000 steps energy minimization using the steepest descent algorithm. The system was equilibrated at 298 K and 1.0 bar, with the MD integrator set at Leap‐frog. The approximate number of frames per simulation was set at 1000, and the simulation time was set to 50 ns.

### 2.9 Immune Simulation

For the prediction of human immune response to the invading particles/foreign materials, the C‐ImmSim server (http://150.146.2.1/C-IMMSIM/index.php) was employed to simulate the immunogenicity of the predicted epitopes. The immune simulator server operates on the principles of innate and humoral responses posed by the invading pathogen, and these are represented as agents [41]. The C‐ImmSim is an agent‐based model that uses machine learning techniques to perform immunological defense mechanisms. The C‐ImmSim algorithm instigates the following protocol: MHC I and II restriction, memory backup, clonal deletion, and antigen presentation (Kwarteng et al. 2020). The vaccine construct (antigens of the *P. falciparum* and antibodies) was uploaded to the C‐ImmSim web server with all parameters set to default. In pursuit of determining the immune response profile of the vaccine administration, three doses were administered at 0, 1, and 2 months, with time steps set at 1, 84, and 168 (a time step equals 8 h in reality). The final time step of the immune simulation was set to 1095 (1 year in reality) to profile the immune response triggered by the vaccine for 1 year.

## 3 Results

### 3.1 Antigenicity Screening of Selected *P. falciparum* Antigens

The five antigenic proteins of *P. falciparum* were obtained from the UniProt protein database (Table 1) and then screened for antigenicity using the Vaxijen v2.0 server with a default threshold of 0.5. All five target proteins had an overall antigenicity at a threshold of > 0.5. This shows that the proteins can elicit an immune response. The three‐dimensional structure of the target proteins (A1, A2, A3, A4, and A5) were generated using AlphaFold v2.0.

**Table 1:** The five highly conserved *Plasmodium falciparum* antigens considered for this study.

Protein tag	Target protein name	UniProt accession number	Antigenicity
A1	Thrombospondin‐related	Q94662	Antigen
	adhesive protein (**PfTRAP**)		
A2	Reticulocyte binding	Q8IFM5	Antigen
	homolog 5 (**PfRH5**)		
A3	Schizont egress antigen‐1	A0A143ZXM2	Antigen
	(**PfSEA**‐1)		
A4	Glutamic acid‐rich protein	P13816	Antigen
	(**PfGARP**)		
A5	Cysteine‐rich protective	Q8IFM8	Antigen
	Antigen (**PfCyRPA**)		

### 3.2 B‐ Cell Epitope Prediction

Bepipred 2.0 and ElliPro pipelines were used in predicting both linear and discontinuous B‐cell epitopes of each of the five target proteins, respectively. The default threshold of 0.5 input on the Bepipred server showed 6, 6, 18, 7, and 10, respective candidates which varied in peptide length. Similar default input set at a threshold of 0.5 and a center position of 4 on the ElliPro server showed an output of 15, 15, 96, 18, and 13 candidates respectively for discontinuous epitopes for each antigen and varied in peptide length.

### 3.3 T‐ Cell Epitope Prediction

The antigens epitopes that were capable of binding specific HLA alleles to elicit T‐cell response against *P. falciparum* were predicted using an IEDB analysis server. Percentile rank < 0.1% was set to select from the large data set predicted, epitopes that have a high binding affinity to specific HLA alleles. All target proteins gave substantial MHC‐I and MHCH‐II epitopes at the < 0.1% cut‐off for the percentile rank.

### 3.4 Assessment of Epitopes

Epitopes were assessed for vaccine construct based on antigenicity, non‐toxicity, non‐allergenicity, and the ability to induce IFN‐γ ‐ for interaction with MHC‐I and MHC‐II. After subjecting the predicted B‐ cell epitopes to verification, 1 epitope from each of the 5 target proteins passed all the assessment properties. For both MHC‐I and MHC‐II, 3 epitopes from 3 antigens (1 each) were found to be ideal for further processing and validation (Table 2).

**Table 2:** Assessment and evaluation of selected B cell, cytotoxic T cells, and helper T cell epitopes from the targeted *P. falciparum* antigens.

B cell epitopes									
Antigen	Epitope number	Peptide	Length	Antigenicity	Allergenicity	Toxicity	IFN‐γ	Alleles	RMSD (Å)
A1	1	MNHLGNVKYLVIVFLIFFDLFLVNGR	26	Probable ANTIGEN	PROBABLE NON‐ALLERGEN	Non‐Toxin	NA	NA	6.36
A2	2	KKTKNQEN	8	Probable ANTIGEN	PROBABLE NON‐ALLERGEN	Non‐Toxin	NA	NA	3.16
A3	3	DEMKEEKEERNEDRGI	16	Probable ANTIGEN	PROBABLE NON‐ALLERGEN	Non‐Toxin	NA	NA	2.43
A4	4	TGQHKPKNATEHGEENLDEE	20	Probable ANTIGEN	PROBABLE NON‐ALLERGEN	Non‐Toxin	NA	NA	7.4
A5	5	NDNNFAE	7	Probable ANTIGEN	Non‐Allergen	Non‐Toxin	NA	NA	0.4

**T cell epitopes (MHC‐I)**									
A1	6	NVIGPFMKAV	10	Probable ANTIGEN	PROBABLE NON‐ALLERGEN	Non‐Toxin	POSITIVE	HLA‐A*68:02	3.079
A2									
A3	7	IYKNPFHHR	9	Probable ANTIGEN	PROBABLE NON‐ALLERGEN	Non‐Toxin	POSITIVE	HLA‐A*33:01	2.78
A4	8	VPLPSPLTDV	10	Probable ANTIGEN	Non‐Toxin	PROBABLE NON‐ALLERGEN	POSITIVE	HLA‐B*51:01	3.28
A5									

**T cell epitopes (MHC‐II)**									
A1	9	YLVIVFLIFFDLFLV	15	Probable ANTIGEN	PROBABLE NON‐ALLERGEN	Non‐Toxin	POSITIVE	HLA‐DQA1*01:01/DQB1*05:01	1.37
A2	10	DDSYRYDISEEIDDK	15	Probable ANTIGEN	PROBABLE NON‐ALLERGEN	Non‐Toxin	POSITIVE	HLA‐DRB3*01:01	8.05
A5	11	HKKFISFFQIVL	12	Probable ANTIGEN	Nonallergenic	Non‐Toxin	POSITIVE	HLA‐DPA1*01:03/DPB1*04:01	2.93

### 3.5 Epitope Stability Studies

The stability of all 11 validated epitopes was determined by running a 100 ns MD simulation and their respective RMSD values were obtained (Table 2). The RMSD value measures the extent of deviation between two overlapping molecular structures [42]. The lower the RMSD value, the narrower the deviation, and vice versa. The least RMSD values among the identified epitopes (for B‐cell epitopes, MHC‐I epitopes, and MHC‐II) of the *P. falciparum* antigens were selected and used in subsequent prediction studies (Table 2).

### 3.6 Structural Properties of the Multi‐Epitope Vaccine Construct

#### 3.6.1 Construction of Vaccine Model

In this process, the construct (255 amino‐acid vaccines) was made by using the selected B‐cell epitopes (a total of five), MHC‐I epitopes (a total of three), and MHC‐II (a total of three) epitopes that met all the filtering criteria. TLR‐4 and TLR‐2 adjuvants (APPHALS and GIINTLQKYYCRVRGGRCAVLSCLPKEEQIGKCSTRGRKCCRRKK) were linked by the EAAAK linker and also attached to the N‐terminal by the EAAAK linker to enhance the immunogenicity of the vaccine. Then, the selected MHC‐II epitopes (a total of three epitopes) were joined together with the GPGPG linker, preceding the last linker after the last MHC‐II epitope, B‐cell epitopes were joined to the chain and were linked by the KK linker. Preceding the KK linker after the last B‐cell epitope, MHC‐I epitopes were joined to the chain and linked together with the AAY linker to get the vaccine candidate sequence, respectively. Earlier works have shown that TLR4 is part of a larger class of toll‐like receptor proteins which function in initializing cascades of immune responses against a foreign agent. TAT sequence was placed downstream of the sequence of the multi‐epitope vaccine construct specifically after the AAY linker and the final multi‐epitope vaccine constructs with 255 amino acid residues have been represented in Figure 3A. With the adjuvants and the TAT sequence respectively positioned at the N‐, and C‐terminal of the vaccine construct, the epitope groups in between (MHC‐I, MHC‐II, and B cell epitopes) were shuffled in order of their placement within the N and C terminal. This gave six variants of the vaccine construct and accordingly named vac1 to vac6 (Supplementary sheet 5).

#### 3.6.2 Population Coverage Analysis

**Figure 2:**
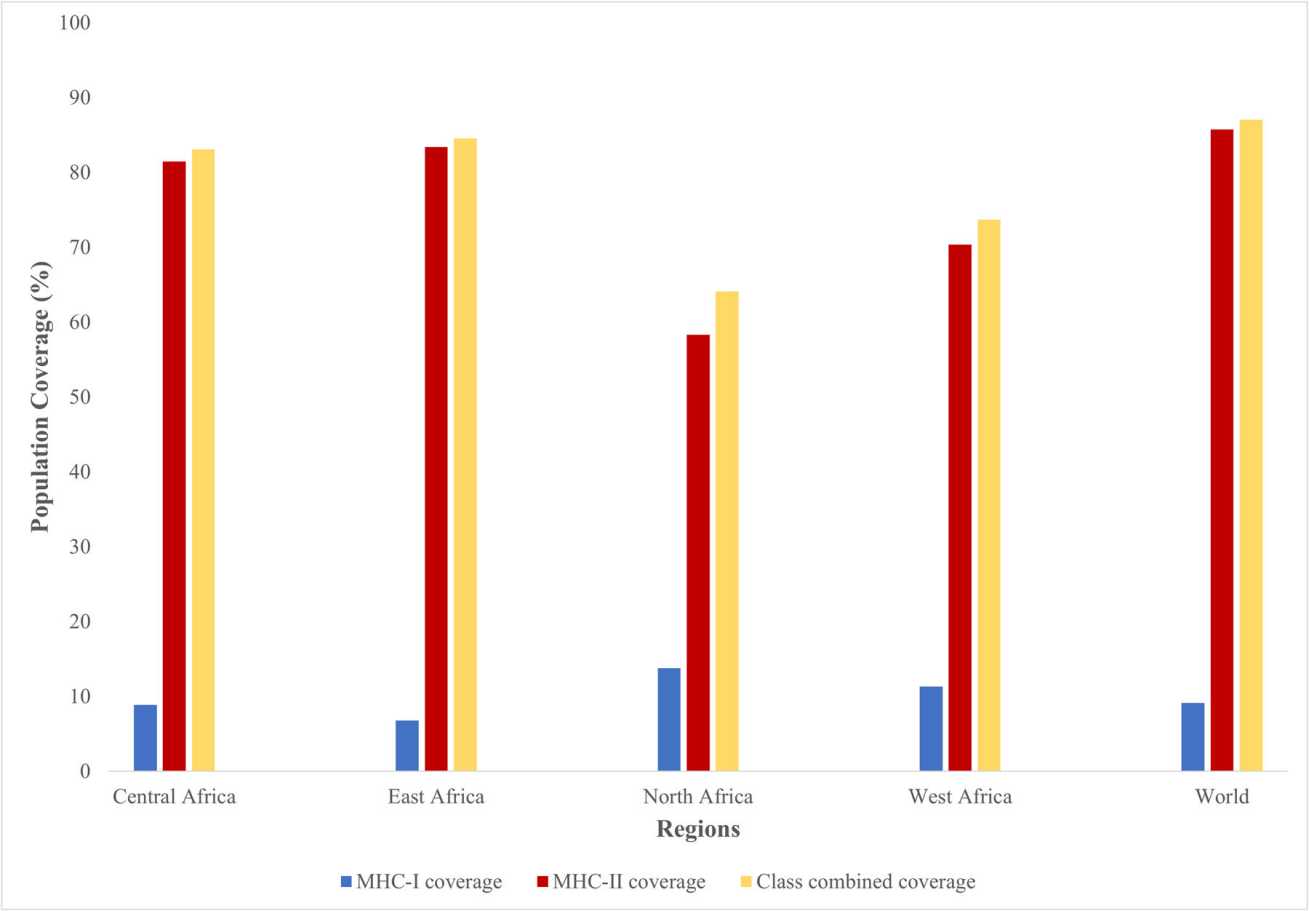
Worldwide, and African population coverage of selected T cell epitopes of the vaccine construct. This graph reveals the fraction of individuals that are predicted to respond to the selected class I, and class II MHC epitopes based on their respective allele frequencies, and their MHC binding profiles.

To ascertain the population coverage of the selected MHC‐I and MHC‐II epitopes of the vaccine construct, we utilized the IEDB population coverage analysis tool. The selected MHC‐I and MHC class II epitopes in the construct can cover 9.17% and 85.76% of the world′s population, respectively. When combined, the world population coverage both MHC epitope classes confer was found to be 87.07%. Similarly in all geographical regions in Africa, MHC‐II epitope coverage was seen to be higher than those of MHC‐I (Figure 2). When used in combination, the African population coverage of MHC‐I and MHC‐II epitopes of the vaccine construct is highest in East Africa (84.52%), followed by Central Africa (83.10%), West Africa (73.71%), North Africa (64.08%). South Africa was excluded from this study because the MHC‐II population output from IEDB population analysis tool came out as zero (Supplementary sheet 4).

##### 3.6.2.1 Modeling, Refinement, and Validation of Vaccine Construct

**Figure 3:**
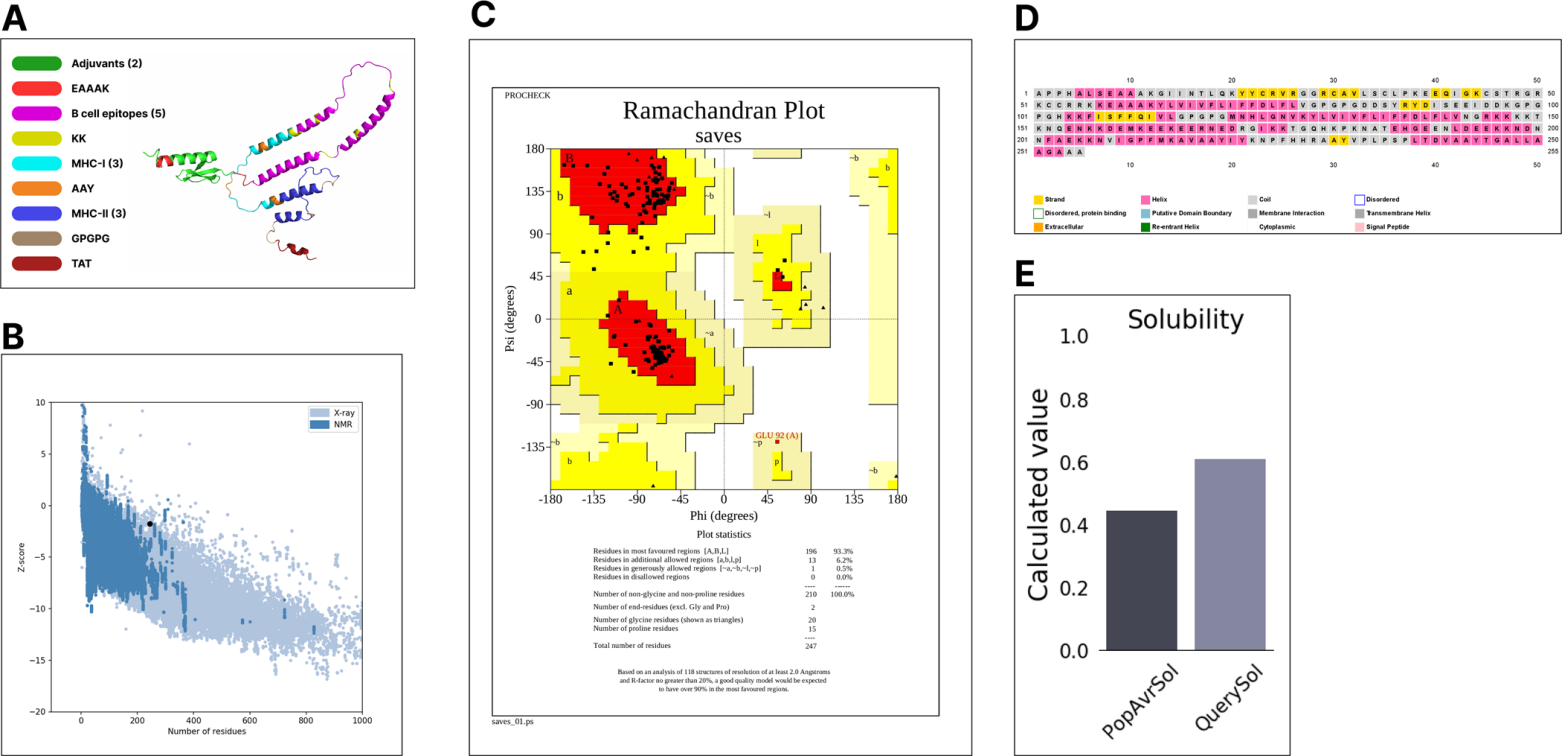
Structural analysis of the designed multi‐epitope vaccine construct. (A) The best generated 3D structure of the vaccine construct from GalaxyRefine color‐mapped by the order of peptide arrangement. (B) Z‐score graph predicted from the ProSA‐server. (C) Ramachandran plot analysis profiling the position of the vaccine′s amino acid residues in the favored, allowed, and disallowed regions. (D) Secondary structure analysis of the vaccine construct predicted by PSIPRED. (E) Generated solubility graph of the vaccine construct from the Protein‐Sol database.

The 3D structures of the respective six vaccine constructs obtained were modeled using AlphaFold v2.0. The highest‐ranked models of the PDB files for each construct were downloaded and inputted into the GalaxyRefine Server for refinement of the 3D structure of the multi‐epitope vaccine model. The vaccine construct needed to be refined to be structurally viable after its development. Comparing results obtained from the 6 refined vaccine variants, vac1 model1 was chosen (Figure 3A) as it had the least RMSD value of 0.624 Å, and 97.6% of its residues were in the Ramachandran favored regions (Table 3). Evaluations of the chosen vaccine model made by the PROCHECK server generated a Ramachandran plot that showed that 96% and 4% of residues are placed in favored, and allowed regions, respectively (Figure 3C). The ProSA z‐score of the best refined model was ‐1.76 (Figure 3B). According to the obtained Ramachandran plot, there were 196 (93.3%) residues in the most favored regions, 13 (6.2%) residues in additional allowed regions, 1 (0.5%) residue in generously allowed regions, and 0 (0%) residues in disallowed regions.

**Table 3:** GalaxyRefine output of the top refined models of all six vaccine variants.

Vaccines	MODEL	GDT‐HA	RMSD	MolProbity	Clash score	Poor rotamers	Rama favored
Vac1	**MODEL 1**	**0.8853**	**0.624**	**1.073**	**2.2**	**0**	**97.6**
Vac2	MODEL 3	0.8471	0.723	1.288	2.7	0	96.4
Vac3	MODEL 2	0.8716	0.656	1.107	3.2	0	99.2
Vac4	MODEL 4	0.8363	0.712	1.23	3.3	0.2	97.4
Vac5	MODEL 2	0.8471	0.812	1.455	3.6	0	95.7
Vac6	MODEL 1	0.8598	0.699	1.025	2.4	0	98.4

*Note:* Bold values are indicate Vac 1 is the strongest overall.

##### 3.6.2.2 Secondary Structure Prediction (Physiochemical Assessment and Protein Stability)

###### 3.6.1.3.1 Physicochemical Properties and Solubility Prediction

To evaluate the secondary properties of the vaccine model, the PSIPRED server was used. The properties that were examined included molecular weight, half‐life, instability index, aliphatic index, grand average of hydropathicity, the total number of negatively and positively charged residues, and iso‐electric point. The Expasy ProtParam server was used to assess these characteristics. The vaccine was made up of 255 amino acids with a molecular weight of 28459.84 Da. Next, it was shown that the vaccine had negatively charged residues (Asp + Glu): 31 and positively charged residues (Arg + Lys): 43 with a theoretical isoelectric point of 9.31. The estimated half‐life of the vaccine was found to be 4.4 h in mammalian reticulocytes (in vitro), > 20 h in yeast (in vivo), and > 10 h in E. coli (in vivo). The instability index was estimated to be 31.89. From this pipeline, an instability index below 40 indicates that the inputted peptide is a stable protein, indicating that the vaccine construct is stable. The aliphatic index was 75.80 indicating that it is thermostable, and the grand average hydropathicity was found to be ‐ 0.516. The negative value indicates that the vaccine construct is hydrophilic and can form favorable interactions with surrounding water molecules. The result from the Protein‐Sol server further corroborates the solubility of the vaccine construct. A detailed summary of the vaccine′s physicochemical properties is shown in Table 4. The PSIPRED server was used for predicting the secondary structure properties of the best refined final model of the designed vaccine (Figure 3E). The refined vaccine model was found to contain 48.63% α‐helix (124 residues), 9.80% β‐strand (25 residues), and 41.57% random coil (106 residues), as seen in Figure 3D.

**Table 4:** Generated physicochemical properties of the vaccine construct. Except stated otherwise, all values were predicted from the ProtParam server.

Characteristics	Finding	Remark
Number of amino acids	255	—
Molecular weight	28459.84 Da	—
Theoretical pI	9.31	Basic
Theoretical pI (Protein‐Sol)	9.91	Basic
Predicted solubility (Protein‐Sol)	0.611	Soluble
Chemical Formula	C_1281_H_2021_N_355_O_361_S_9_	—
Extinction coefficient (at 280 nm) ‐ measured in water	15275 M^‐1^cm^‐1^	—
Estimation half‐life (mammalian reticulocytes, in vitro)	4.4 h	—
Estimated half‐life (yeast cells, in vivo)	> 20 h	—
Estimated half‐life (*E. coli*, in vivo)	> 10 h	—
Aliphatic index	75.80	Thermostable
Instability index	31.89	Stable
Grand average of hydropathicity (GRAVY)	‐ 0.516	Hydrophilic

#### 3.6.3 Molecular Docking Studies

**Figure 4:**
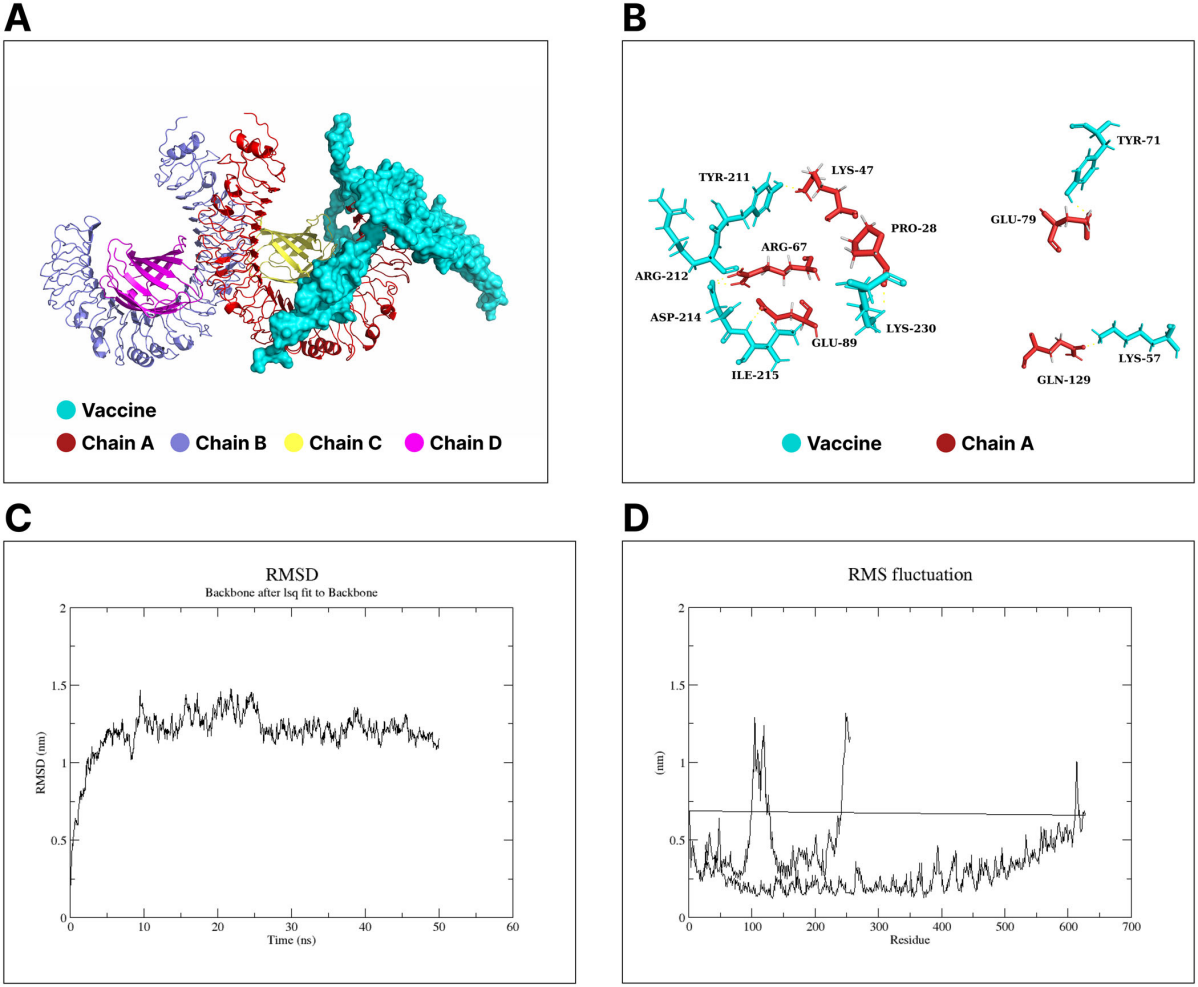
Molecular docking studies and dynamics simulation of the TLR4‐vaccine complex. (A) The best docked pose of the TLR4‐vaccine complex obtained from ClusPro. TLR4 is seen to have four chains: chain A (red), chain B (stale blue), chain C (yellow), and chain D (magenta), with the vaccine showing strong association with chain A of TLR4. (B) Polar interactions between residues of TLR4's chain A and residues of the vaccine construct. (C) The RMSD and (D) RMSF plots of the interaction zone of the TLR4‐vaccine complex.

The binding affinity between TLR4 and the vaccine construct was evaluated through molecular docking studies. The server generated 30 poses (clusters) and calculated the number of members and corresponding lowest energy for each pose. Comparing all clusters revealed that cluster 0 had the most members (34) with the lowest energy of ‐ 1262.3 and was considered for further analysis (Supplementary sheet 7). Visualization of the selected docked complex revealed that the vaccine candidate formed significant interactions with chain A of the TLR4 (Figure 4A). Residues of vaccine construct (Lys57, Tyr71, Tyr211, Arg212, Asp214, Ilu215, and Lys230) were found to form polar interactions with residues (Gln129, Glu79, Lys47, Arg67, Arg67, Glu79, and Pro28) of chain A of TLR4, respectively (Figure 4B).

##### 3.6.3.1 Molecular Dynamic Simulation to Assess Conformational Stability and Flexibility

The RMSD is defined as the distance between the backbone carbon alpha atoms of the stacked proteins [42]. Owing to the limitation of the WebGro online MD simulation pipeline which only allows for up to 150,000 atoms in the total system (including solvent), chain A of TLR4 in complex with the vaccine construct (extracted from the 3D structure of cluster 0) was subjected to MD simulation studies to characterize the stability of the interaction zones of the complex. The backbone carbon atoms' RMSD of the TLR4 chain A‐vaccine complex showed a gradual increase in structural deviation between ~0.2 and 1.5 nm (approximately up to 28 ns). However, after 28 ns, the RMSD value of the complex became stable with a value of approximately 1.25 nm (Figure 4C **)**. The dynamics of the RMSD profile over the 50 ns simulation revealed that the complex exhibited a stable binding interaction at the interaction zones.

The root mean square fluctuation (RMSF) was used to assess the flexibility of the docked complex. Residual fluctuations were seen within residues ~ 90–150, and ~ 240–255 of the vaccine component of the complex while there were slight fluctuations at the C‐terminal of the receptor starting from residue 500. The overall RMSF profile of the complex indicated a relatively lower RMSF value < 1.5 nm, which further corroborates to the stability of the vaccine‐TLR4 interaction zone (Figure 4D).

### 3.7 In‐Silico Immune Simulation

**Figure 5:**
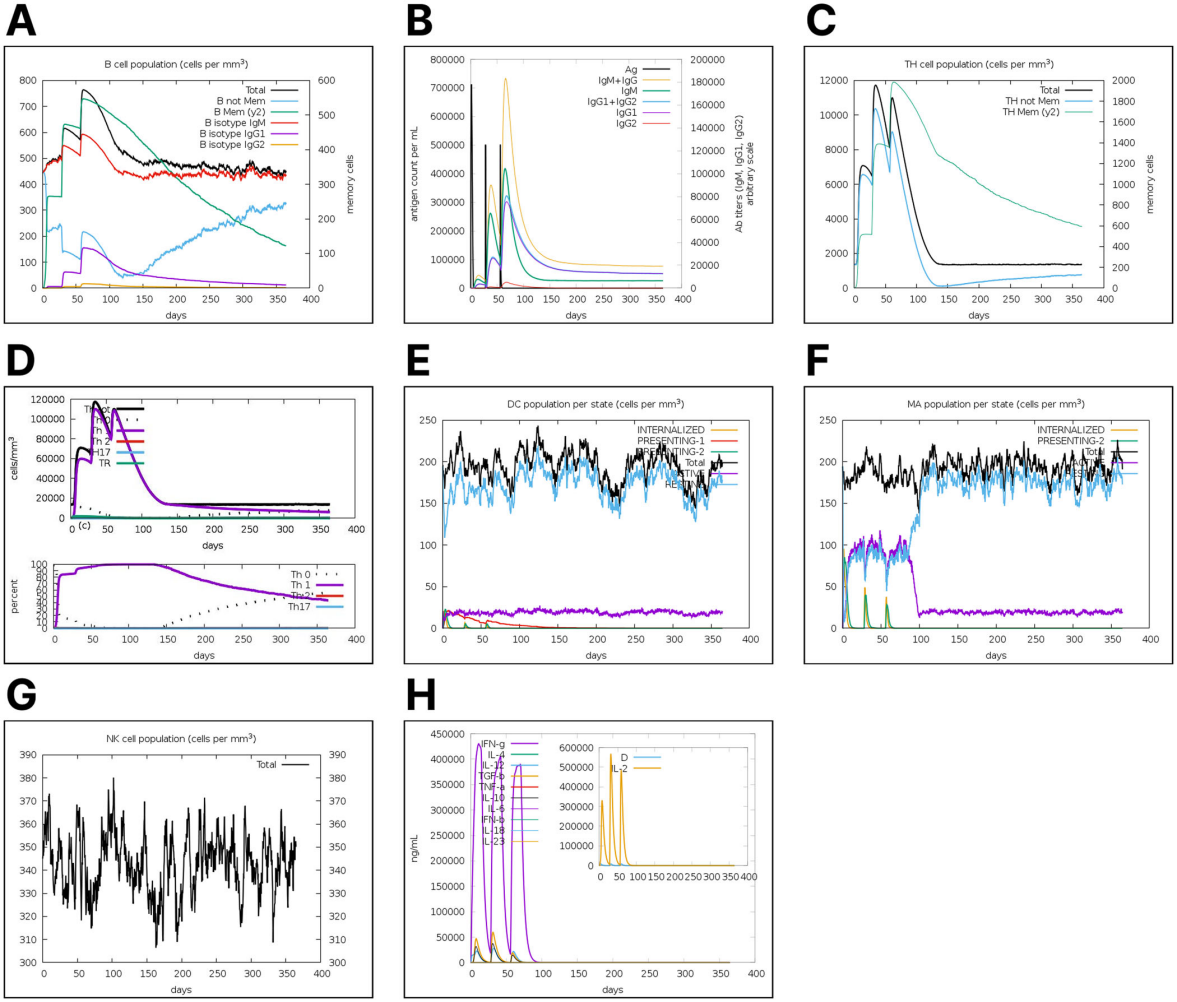
Simulation of the immune response upon vaccine administration. (A) B cell population profile (cells per mm3). Black line indicates total B cell population. Blue line shows non‐memory B cell population (shown as B not Mem in graph). Green line represents a scale of memory B cell populations [BM (y2)]. Red line reveals B isotype subpopulations expressing IgM, purple line indicates B isotype subpopulation expressing IgG1, and yellow line shows B isotype populations expressing IgG2. (B) Immunoglobulin concentration relative to antigen clearance. Black line signifies antigen count per mL. Yellow line shows the combined IgM and IgG antibody titers. Green line reveals IgM levels. Blue line indicates combined IgG1 and IgG2 antibody peaks. Purple line shows IgG1, and orange line indicates IgG2 titers. Besides total antibody count (black line), all antibody peaks are set to the C‐Imm Server arbitrary scale. (C) Helper T (Th) cell population (cells per mm3). Black line shows total helper T cell populations. Blue line represents non‐memory helper T cells (shown as Th not Mem in graph). And green line indicates memory helper T helper populations [defined as Th Mem (y2) in graph]. (D) Specific helper T (Th) cell subtypes induced. Black line represents Th tot (total Th cell population). Dotted black line shows Th 0 (naive T helper cells). Purple lines represent Th 1 cells (type 1 Th cells). Red line signifies Th2 (type 2 Th) cells, blue line, TH17 (type 17 Th) cells, and green line indicates TR cell populations. (E) Dendritic cell (DC) population per state (cells per mm^3^). Yellow line shows DCs in the internalization state (described as internalized). Red line indicates type 1 conventional dendritic cells (defined as Presenting‐1 in graph). Green line displays the profile of type 2 conventional dendritic cells (defined as Presenting‐2 in graph). Black line shows the total dendritic cell populations. Purple reveals the active DCs, while the blue line represents resting DCs. (F) Macrophage (MA) population profile upon vaccine administration. Yellow lines indicate macrophages in the internalized state. Green line shows presenting‐2 macrophages, and black line signifies total macrophages. Purple line displays active macrophages, and blue line represents resting macrophages. (G) Natural killer (NK) cell response. Black line indicates total NK cell population per mm3. (H) Concentration profile of released cytokines. Thick Purple peaks indicate interferon gamma (IFN‐g). Thick green line shows IL‐4. Thick blue line (left legend) represents IL‐12, thick yellow line (left legend) shows TGF‐β, and thick red line indicates TNF‐α. Black line indicates IL‐10. Thin purple line shows IL‐6, thin green line represents IFN‐β, thin blue line shows IL‐18, and thin yellow line (left) represents IL‐23. For the graph to the right, blue line signifies generic danger signal (D), while the yellow peaks (right legend) represents IL‐2.

To evaluate how the immune system responds to the vaccine construct, an in‐silico prediction of the immune response was conducted. The immune simulation was performed by employing the C‐ImmSim web server which can simulate both humoral and cell‐mediated immune response in‐silico. After three doses of injections with a 28‐day interval, the immune response profile of the designed vaccine is shown in Figure 5. Administration of the vaccine revealed an increase in IgM, and IgG1 and G2 secreting memory B cell population (Figure 5A). The combined IgM + IgG secreted titer remained at about 750,000 count/mL; the IgM titer alone was calculated to be around 400,000 count/mL, and the combined IgG1 + IgG2 titer was about 300,000 count/mL. These data show that the titer of secreted immunoglobulins increased after the injection of the designed vaccine, with a marked decrease in the antigen concentration (Figure 5B). Further, there was a significant increase in memory helper T (Th) cells (Figure 5C), with the Th‐1 subtype being more predominant in the Th cell population (Figure 5D). It was observed that the memory B and Th cells elicited slowly declined but maintained a significant population size till approximately day 100 after the first vaccine administration (Figure 5A,C,D). The innate arms of the immune system were considerably induced by the vaccine′s presence. Specifically, dendritic cells (DC), macrophages (MA), and natural killer (NK) cells were elicited by the proposed vaccine administration (Figure 5E,F,G). Analysis of interleukins (IL) and cytokines productions showed high titers of interferon gamma (IFN‐γ), IL‐2, and transforming growth factor β (TGF‐b) were seen to have been induced after the vaccine administration with an overall insignificant generic danger signal (D) value (Figure 5H).

## 4 Discussion

This study sought to predict a multi‐epitope vaccine candidate against *P. falciparum*, the causative organism of malaria, by identifying antigenic peptides from five highly conserved peptides from the organism. The multi‐epitope vaccine candidate derived from this study is constituted of three CD8^+^ T‐cell epitopes, three CD4^+^ T‐cell epitopes, and five B‐cell epitopes, all concatenated by suitable linkers. This vaccine is intended to train the immune system to recognize highly conserved peptide regions of *P. falciparum*, which would translate to a fast response by the immune cells against the malaria‐causing microorganism and could reduce the immune evasive effect of antigenic variation in the parasite.

In recent times, immunoinformatics‐guided predictions of epitope‐based subunit vaccines have been widely used as a first line of vaccine development against a number of infections [43, 44, 45]. This is, in part, due to its relatively fast and cost‐effective approach. Using this technique, helper T‐cell, cytotoxic T‐cell, and B‐cell epitopes were predicted from five highly conserved *P. falciparum* antigens. The identified epitopes were further predicted to be nontoxic, nonallergenic, and antigenic, with the predicted T cell epitopes being able to elicit IFN‐γ response. These epitopes were identified respectively from *P. falciparum*′s thrombospondin‐related adhesive protein (PfTRAP), reticulocyte binding homolog 5 (PfRH5), schizont egress antigen‐1 (PfSEA‐1), glutamic acid‐rich protein (PfGARP), and cysteine‐rich protective antigen (PfCyRPA). These highly conserved *P. falciparum* antigens have been used, either independently or in complex with other antigens, for the development of malaria vaccines and are experimentally known to reduce parasitic burden [46, 47, 48, 49, 50]. However, due to antigenic polymorphism and the complexity of the multistage life cycle of the apicomplexan, vaccine development against malaria has been challenging [51]. Therefore, the combination of these conserved antigens for a multi‐epitope vaccine can be seen as an ideal explorative avenue for vaccine development.

An administered vaccine must increase the population of memory B and T cells trained to recognize specific antigens so as to trigger a rapid response to future pathogenic invasions having similar antigenic signatures to previously exposed antigens [52]. It has been well documented that during *P. falciparum* infections, high levels of *Plasmodium*‐specific CD4^+^ T cells correlate to a state of protective immunity preceding natural exposure to the pathogen, or anti‐malarial vaccination [53, 54, 55]. In addition, effector Th1 cells and interferon‐gamma (IFNγ) are known to contribute to host *Plasmodium* protection, especially at the blood‐stage infection phase [56]. Results from our immune simulation studies revealed that the vaccine construct, upon administration, can increase the population of active and memory CD4^+^ Th1 cells, and IFNγ concentrations within the first 7 days after the first vaccine administration. This result corroborates with findings from a human study in the Gambia that established a positive association between IFNγ‐secreting *P. falciparum‐*specific CD4^+^ T cells and anti‐malarial protection [57]. It should also be noted that our population coverage study revealed that the majority of our selected MHC‐II epitopes for the vaccine construct, needed to elicit CD4 + T cells responses, had a wider population coverage in Africa, and globally as compared to that of MHC‐I epitopes. This gives an indication that our proposed vaccine, upon administration, can induce CD4^+^ T cell‐mediated *Plasmodium* protection on a wide population coverage in Africa, and the world at large. Antibodies against *P. falciparum* that prevent blood‐stage infection are crucial players of protective immunity [58]. The immune simulation study revealed an increase in activated and memory B cell population, ultimately resulting in the increase in peripheral blood B isotype immunoglobulin M, and G1 (i.e., IgM, and IgG1). It has been documented that IgG is important for the protection from high parasitaemia clinical symptoms of malaria [59, 60]. Similarly, Dobaño et al. [61] observed that the cytophilic subclass IgG1 contributes to malaria protective immunity [61]. Immunoglobulin M (IgM) has also been reported to contribute to malaria immunity through its long‐lived antibody response against blood‐stage malaria parasites [58].

The simulated immune response also revealed a heightened activation of certain arms of the immune system. Primarily, there was an increase in dendritic cells (DC), macrophages, as well as natural killer cell populations, respectively. The importance of dendritic cells in antimalaria protection has been documented. DCs are professional antigen‐presenting cells that play crucial roles in initiating the adaptive immune response against a myriad of pathogens, including *Plasmodium* [62, 63, 64]. Dendritic cells presenting *Plasmodium* sporozoite antigens in murine models are known to initiate protective immune responses against malaria by activating specific CD4+ and CD8 + T cells [65]. The role of macrophages during malaria manifestation has been debatable. From one standpoint, they confer anti‐malarial protection to the host by inducing antibody‐dependent cell inhibition, phagocytosis, and cytokine production. From a different standpoint, they may contribute to enhancing malaria infection and related complications [66]. *P. falciparum‐*activated natural killer cells have been seen to respond to malaria parasites through natural cytotoxicity and could inhibit parasite growth through antibody‐dependent cellular cytotoxicity mechanisms. In Addition, they are known to partner with myeloid cells to release inflammatory cytokines interferon‐gamma for *Plasmodium* clearance [67].

Toll‐like receptors (TLRs) form the foundation of the innate immune responses through recognition of invading pathogens upon their activation [68, 69]. Located at the external plasma membrane layer, the toll‐like receptor 4 (TLR4) is a pattern recognition receptor capable of recognizing *P. falciparum* antigens [70]. Assessing the docked TLR4‐vaccine complex, it can be inferred that the complex formed energy‐favored interactions based on the lowest energy score (‐1262.3) and the cluster size (34) obtained from the docking study carried out. The structural stability of the complex was investigated through molecular dynamics simulations. The RMSD of the complex increased slightly until ~28 ns of simulation time before achieving an equilibrium that reflects an average RMSD that remained at 1.25 nm. The low average RMSD magnitude obtained indicates strong conformational stability achieved by the interacting regions of the TLR4‐vaccine complex [71]. The root mean square fluctuations (RMSF) parameter helps indicate how amino acid residues of proteins contribute to a stable conformation for a protein‐ligand (in this case, TLR‐vaccine) complex [71]. The root mean square fluctuations (RMSF) of the amino acid residues in the complex indicate the levels of flexibility each residue has. Thus, residues displaying higher RMSF levels indicate increased flexibility, implying their increased ability to form strong interactions within the complex [71]. Interestingly, the RMSF plot indicates distinct fluctuations at approximately residue 70– 50, and 210–255 of the vaccine component in the complex, and at residues 1– 70, and beyond residue 500 of the receptor portion of the complex. These zones of fluctuations fall within the positions of the vaccine, and TLR4 residues involved in the identified polar interactions of the complex. This further indicates the structural stability of the interacting regions of the vaccine and TLR4.

The effectiveness of a multi‐epitope‐based subunit vaccine is, in part, influenced by its physicochemical attributes. Typical examples include hydrophilicity and hydrophobicity of the construct. The negative GRAVY score obtained from ProtParam Server for the vaccine construct identifies the vaccine as hydrophilic. Also, the predicted solubility obtained from the Protein‐Sol server classifies the construct as soluble. Based on the hydrophilicity and solubility findings, it can be inferred that the vaccine construct can form strong interactions with water molecules which could positively influence the vaccine′s movement within the aqueous milieu of the human vasculature. Another physicochemical parameter that contributes to vaccine effectiveness is its molecular weight. A vaccine with a high molecular weight of approximately 40–50 kDa has a higher chance of its molecular constituents being directly transported from sites of injection to draining lymph nodes to develop active immune responses [72, 73, 74]. In this line of reasoning, the vaccine′s predicted molecular weight of 27.87 kDa could suggest that for a more elevated active immune response to be induced by the construct, macromolecular conjugates should be considered in the vaccine′s constitution to increase the effective hydrodynamic size of this proposed vaccine [72]. The structural construct of a vaccine design must be seen as stable. From the ProtParam server, the instability index obtained predicts the vaccine construct to be stable. Further, the low RMSD magnitude of 0.653 Å of the refined vaccine construct supports the stability of the construct. Further, the aliphatic index generated for the vaccine predicts the vaccine to be thermostable. These findings collectively indicate that the vaccine is a stable construct.

It should be stated, however, that this study has potential limitations. Due to set restrictions on the WebGro online MD simulation server, the simulation time was set at 50 ns, which represents the maximum allowed simulation time on their server. Moreso, the WebGro server was unable to run MD simulations for the vaccine construct complexed with the complete TLR4 molecule due to the total number of atoms limit exceeded in the initial PDB complexed file inputted. Hence, only the interacting chains of the TLR4‐vaccine complex were extracted and inputted for the MD simulation study. To add, regardless of the MHC epitopes selected percentile rank cut‐off of < 0.05% to 0.1%, which hinted a high‐affinity binding between epitope and MHC receptors, molecular docking studies of the selected T cell epitopes and their respective MHC receptors were not carried out in this study. This study, therefore, requires further experimental validation of the proposed vaccine′s immune reinforcement by synthesizing and characterizing the predicted multi‐epitope construct, and performing in vitro and in vivo immunogenicity validations.

## 5 Conclusion

Malaria continues to remain a major contributor to high mortality and morbidity rates in African populations. In this study, we employed immunoinformatics to identify ideal epitopes from five highly conserved *P. falciparum* antigens which were used to construct a multi‐epitope subunit vaccine against malaria‐causing *P. falciparum*. Based on the results, the nontoxic, nonallergic, and highly immunogenic nature of the vaccine construct suggests its potential to induce cellular and humoral immune responses during malaria manifestations. Despite the *in silico*‐based profiled effectiveness of the proposed vaccine, the immunogenic ability of the vaccine requires experimental validation.

## Author Contributions

**Amma Aboagyewa Larbi:** conceptualization, methodology, writing – original draft, writing – review and editing, investigation. **Caleb Mensah:** conceptualization, writing – review and editing, writing – original draft, visualization. **Rebecca Korankye:** methodology, data curation, writing – review and editing. **Andrew Appiah Darkwah:** methodology, writing – review and editing. **Esther Boakye Agyemang:** methodology, writing – review and editing. **Isaac Yao Ayikpah:** methodology, writing – review and editing. **Alexander Kwarteng:** writing – review and editing, methodology.

## Conflicts of Interest

The authors declare that the research was conducted in the absence of any commercial or financial relationships that could be construed as a potential conflict of interest.

## Transparency Statement

The lead author Amma Aboagyewa Larbi affirms that this manuscript is an honest, accurate, and transparent account of the study being reported; that no important aspects of the study have been omitted; and that any discrepancies from the study as planned (and, if relevant, registered) have been explained.

## Supporting information

Supplementary Sheets Malaria Vaccine.

## Data Availability

The data that support the findings of this study are openly available in Gene Expression Omnibus at https://www.ncbi.nlm.nih.gov/gds.
